# Solute carrier protein family: physiological functions, disease associations, and therapeutic potential in immune-related disorders

**DOI:** 10.3389/fimmu.2025.1671706

**Published:** 2025-12-05

**Authors:** Peiyan Li, Chen Liu, Yewei Niu, Peitao Wu, Lixuan Liu, Jiamin Jin, Jinfeng Yang

**Affiliations:** 1 Department of Immunology, Guilin Medical University, Guilin, China; 2 Key Laboratory of Tumor Immunology and Microenvironmental Regulation, Guilin Medical University, Guilin, China

**Keywords:** solute carrier, transplantation immunity, immune response, transplant rejection, cell metabolism

## Abstract

The Solute Carrier Protein Family (SLC) is responsible for the uptake and transport of a variety of substances across the cell membrane. It plays a central role in maintaining the stability of the intracellular environment through participation in metabolic processes and the transport of drugs and toxins. The highly tissue-specific expression of SLC proteins endows them with potential applications in disease treatment and drug development. Transplant immune reactions are a major challenge in the field of organ transplantation, as graft rejection is a key factor determining the success of transplantation and long-term organ survival. SLC proteins are increasingly drawing attention for their roles in modulating immune responses, influencing transplant immune tolerance, and controlling graft rejection. By regulating the metabolism and function of immune cells, SLC proteins affect the formation and tolerance of transplant immune responses. Among them, 7 SLC proteins are “validated targets” with approved or phase III drugs, 9 are “candidate targets” in active clinical trials, and 14 remain “potential targets” supported by genetic and pre-clinical evidence. This article elucidates the functions of SLC proteins in transplant immunology, inflammation and autoimmune diseases, tumor immunology, metabolic diseases, and neurological diseases, as well as the new targets and strategies for treating these diseases that SLC proteins provide.

## 1 Introduction

The Solute Carrier Protein Family (SLC) is one of the largest families of membrane transport proteins, comprising 458 transporters divided into 65 families (1). These proteins are widely distributed in cell membranes and organelle membranes, responsible for transporting diverse small molecules and ions across the membrane to maintain cellular homeostasis. SLC transporters typically fold into a 10–12 transmembrane α-helical bundle whose substrate-binding site is buried within the bilayer as an amphipathic cleft facing either the extracellular or intracellular milieu. An alternating-access mechanism—driven by substrate-induced tilting or rotation of TM helices—switches the cleft between outward-open and inward-open conformations to accomplish vectorial transport across the membrane. SLC proteins play a crucial role in nutrient uptake, metabolic regulation, and waste expulsion (1). Moreover, they are significantly implicated in various diseases, with dysfunction of certain SLC proteins closely associated with metabolic disorders, cancer, and immune-related diseases. Research indicates that at least 80 SLC proteins are linked to human metabolic diseases, including obesity, type 2 diabetes (T2D), non-alcoholic fatty liver disease (NAFLD), and insulin resistance-related metabolic disorders (2–4). Compared to other membrane protein families, the functions and regulatory mechanisms of SLC membrane proteins are not fully understood, with only a few members identified as drug targets (2, 4). Recently, research targeting the SLC protein family for therapeutic purposes has gradually increased, with small-molecule inhibitors or activators of SLC proteins being developed to treat various diseases, such as diabetes, NAFLD, and certain types of cancer. Jnana Therapeutics Inc. is currently developing a small-molecule inhibitor of SLC6A19 to reduce plasma phenylalanine levels, which is undergoing phase I clinical trials (5).

SLC transporters possess four major biological functions. Mediating the uptake and transmembrane transport of nutrients or energy sources required for vital activities. Participating in the absorption of ions or trace nutrients in the body. Regulating the transmembrane transport and signal transduction of neurotransmitters. Working together to transport and excrete drugs, toxins, and metabolic waste products (1, 6).

In immune-related diseases, abnormal expression or dysfunction of SLC proteins may lead to metabolic disturbances in immune cells, thereby impairing immune responses. Some SLC proteins are critical for regulating the activation, proliferation, and cytokine secretion of immune cells. Dysfunction in these processes may trigger autoimmune diseases and affect the efficacy of immune therapies. The SLC protein family holds great potential for the treatment of immune-related diseases, with future research focusing on the specific mechanisms of SLC proteins in immune metabolism and the development of novel therapeutic agents targeting SLC proteins (1, 7). In this review, we aim to analyze the roles of the SLC family in various immune diseases, providing new insights for the treatment of these diseases using SLC proteins. To intuitively compare the translational potential of SLC members, we have selected 10 “priority druggable targets” and summarized them in **Table 1**. Priority druggable targets.

**Table 1:** Priority druggable targets.

SLC protein	Key immune-related diseases	Mechanism of action	Highest development stage	Key references
SLC15A3	Ulcerative colitis, ankylosing spondylitis, SLE; viral infections (HSV-1)	Proton-coupled import of bacterial/viral peptides into endosomes → triggers NOD2/TLR signaling and IL-1β/IFN-α production	Pre-clinical PCC (small-molecule inhibitor RES-15A3; EU-IMI consortium)	(8, 9)
SLC15A4	Systemic lupus erythematosus, Sjögren’s syndrome, inflammatory bowel disease	Controls TLR7/9 endosomal localization and mtDNA homeostasis; regulates pDC-derived IFN-α	Pre-clinical PCC (Daiichi-Sankyo/RESOLUTE quinolone series)	(10)
SLC19A (RFC1)	Rheumatoid arthritis; tumor immunity (cGAMP antagonism)	Transports immunostimulatory cyclic dinucleotides and MTX; dampens cGAS–STING axis	Marketed drugs repurposed—methotrexate, pemetrexed	(11)
SLC22A4 (OCTN1)	Crohn’s disease, psoriatic arthritis	Transports ergothioneine/butyrobetaine; modulates Th17 differentiation and oxidative stress	Marketed drugs repurposed—metformin, imatinib are known inhibitors	(12)
SLC22A5 (OCTN2)	Crohn’s disease, psoriatic arthritis, AS risk allele	Carnitine uptake → regulates FAO and memory T-cell survival	Repurposed β-lactam antibiotics validated as inhibitors	(13)
SLC46A2	Psoriasis, atopic dermatitis	Transports muramyl dipeptide → activates NOD2; MTX anti-inflammatory activity partly via transporter blockade	Pre-clinical PCC (optimized MTX derivatives; Bharadwaj 2023)	(14)
SLC6A8	Inflammation-associated colorectal cancer, sepsis	Creatine import → skews macrophages toward M2 phenotype; suppresses pro-inflammatory cytokines	Pre-clinical (dual GSK-3β/CRT inhibitor GSK2837808A)	(15)
SLC7A5 (LAT1)	Autoimmune encephalomyelitis, solid-tumor immune evasion	Large neutral amino-acid transport → mTORC1 activation; controls Treg/Th17 balance	Phase I oncology agent (JPH203/KY-003 small-molecule inhibitor)	(16)
SLC7A11 (xCT)	Neuro-inflammation, acute lung injury, ferroptosis-linked immune disorders	Extracellular cystine uptake → GSH synthesis; regulates macrophage ferroptosis and IL-1β release	Phase II oncology (sasikrastat, erastin analogues); multiple marketed drugs (sulfisoxazole) inhibit	(17)
SLC51A/B (OSTα/β)	Primary biliary cholangitis, IBD	Bile-acid efflux → modulates FXR–TGR5 immuno-metabolic axis; suppresses NLRP3 inflammasome	Pre-clinical (FXR agonist–OST conjugate “Tro-Ost”)	(18)

Only include targets that meet all of the following criteria:

1. Functional-immunophenotype confirmed by gene editing or clinical genetics;

2. High-affinity tool compound or approved/marketed drug already available for targeting;

3. Development stage ≥ pre-clinical candidate (PCC) optimization.

Rank by prioritized druggability.

### 1.1 Content filtering and evidence-grading criteria

To balance review depth with readability, we selected SLC proteins for focused discussion in each disease section according to a three-tier scale:

Tier A (validated):

① Human genetic evidence (GWAS P < 5×10⁻^8^ or rare mutation)

② Functional studies in primary immune cells or conditional knockouts

③ Marketed or Phase III small-molecule or antibody

Tier B (candidate):

① + ② + tool compound in Phase I/II

Tier C (potential):

① + ② but no clinical-stage molecule

## 2 The functions of SLC proteins in immune cells

### 2.1 Regulating the metabolism of immune cells

SLC proteins can regulate the metabolic levels of immune cells by transporting nutrients such as glucose, amino acids, and lipids, thereby influencing their functions (19, 20). For example, glucose transporters such as GLUT1 and GLUT3 increase glucose uptake upon T cell activation, promoting aerobic glycolysis and thereby maintaining the activation and differentiation of T cells. Lactate transporters such as SLC16A1 (MCT1) and SLC15A2 help T cells take up lactate in inflamed areas, regulating intracellular metabolic homeostasis. In dendritic cells (DCs), SLC transporters influence their maturation and functional performance by regulating the transport of amino acids and metal ions, thereby modulating innate and adaptive immune responses (19). Within the population of macrophages, SLC transporters regulate their functions, such as in M1-type macrophages, by influencing cellular metabolism and mitochondrial activity. These research findings indicate that SLC transporter proteins are not only key regulators of cellular metabolism but also adjust the intensity and direction of immune responses by influencing the metabolic reprogramming process of immune cells (21). SLC transporters can regulate signaling pathways in immune cells by transporting specific substrates. Besides transporting folate and antifolate drugs, SLC19A1 is also involved in the transport of cyclic dinucleotides (CDNs). CDNs are key immune signaling molecules that can activate the STING pathway, triggering a broad range of immune responses (20, 22). In T cells, the complex formed by SLC7A5 and SLC3A2 can activate the mTORC1 signaling pathway by transporting leucine, thereby regulating the metabolism and function of T cells (20).

### 2.2 Influence the maturation and differentiation of immune cells

SLC transporters also play a crucial role in the maturation and differentiation of immune cells. In DCs, SLC11A1 (NRAMP1) is highly expressed and involved in antigen presentation and immune regulation (20). Similarly, SLC11A1 is highly expressed in macrophages, where it participates in iron ion transport and is essential for the phagocytic and bactericidal functions of macrophages (20). Recent studies have found that SLC11A1 expression is significantly elevated in peripheral blood mononuclear cells of Alzheimer’s disease (AD) patients and is closely associated with antigen processing and presentation pathways. This suggests that SLC11A1 may participate in the immune regulation of neuroinflammation and neurodegeneration by influencing the antigen-presenting function of dendritic cells (DCs) (23). SLC transporters also play an important role in immune regulation, especially in tumor immunotherapy. For example, the transporters SLC13A3 and SLC4A4—among others—will be discussed in detail in the subsequent sections.

Given the crucial roles of SLC transporters in immune cells and tumor cells, they have become potential targets for immunotherapy. By targeting SLCs in multiple immune cells (such as T cells, DCs, NK cells, and macrophages), it is possible to enhance the efficacy of immune cells and increase the effectiveness of anti-tumor immunity (19, 20). Additionally, by modulating the functions of SLC transporters, more precise drug treatment strategies can be achieved, especially during the period of immunosuppressive therapy following organ transplantation (20).

## 3 The roles of SLC family in various immune diseases

### 3.1 The role of SLC proteins in inflammation and autoimmune diseases

SLC proteins exert dual roles in inflammation and autoimmunity by modulating immune cell metabolism, amino acid/ion transport, and signaling pathways. Targeting SLC15A4, SLC26A4, SLC1 family members, and OCTN transporters represents a promising therapeutic strategy for inflammatory and autoimmune disorders.

SLC proteins play crucial roles in modulating inflammatory responses and immune cell functions. SLC15A4 modulates Toll-like receptor (TLR) signaling in plasmacytoid dendritic cells (pDCs), influencing inflammatory responses, and its inhibitors exhibit anti-inflammatory potential (24). SLC26A4 regulates macrophage autophagy and NLRP3 inflammasome activation, serving as a potential therapeutic target for inflammatory modulation (25). SLC22A5 and SLC30A8 are critically involved in asthma-related airway inflammation and immune cell function, with SLC22A5 also mediating drug absorption mechanisms in bronchial epithelial cells (25). The SLC7 family, including SLC7A1 (CAT1), SLC7A2, SLC7A5/SLC7A7/SLC7A11, regulates amino acid transport in T cells and macrophages, modulating inflammatory responses (26, 27). OCTN1/OCTN2 (SLC22 family) transport anti-/pro-inflammatory substrates, with genetic polymorphisms linked to Crohn’s disease and rheumatoid arthritis, and their expression is regulated by inflammatory cytokines (28–31).

In autoimmune diseases, SLC proteins also have significant implications. SLC15A4 is a key pathogenic factor in systemic lupus erythematosus (SLE), with dysfunction correlating with disease progression (24). EAAC1 (SLC1A1) has reduced expression in multiple sclerosis (MS), impairing glutamate clearance and exacerbating neuroinflammation and excitotoxicity (26, 32). SLC1A2 (GLT1) and SLC1A3 (GLAST) are downregulated in MS, leading to synaptic glutamate accumulation and driving neuronal damage (33–35). SLC1A5 (ASCT2) deficits in intestinal epithelial cells impair antimicrobial peptide synthesis, worsening inflammatory bowel disease (IBD) (26, 27, 36), while reduced expression in psoriasis and obesity disrupts skin and T cell function (26, 33). SLC2A4/SLC9A3/SLC11A1 regulate glucose, iron, and zinc transport in IBD, influencing gut immunity and inflammation (25). The SLC15/SLC46 families transport bacterial peptidoglycans to activate NOD1/2 receptors, contributing to autoimmune pathogenesis (21, 26). Additionally, SLC-mediated cyclic dinucleotide (CDN) delivery activates STING signaling, triggering autoimmune responses (21).

### 3.2 The role of SLC proteins in metabolic diseases

Non-alcoholic fatty liver disease (NAFLD) is closely associated with obesity and type 2 diabetes, and is one of the components of metabolic syndrome. SLC proteins are abundantly expressed in the liver, participating in the transport of various nutrients and metabolites and regulating liver physiological functions. Some SLC transporters have become new targets for drug development. SLC25A1, a specific citrate transporter, has its inhibitor CTPI-2 capable of reducing macrophage infiltration, preventing steatohepatitis, and ameliorating obesity induced by a high-fat diet. Inhibition of SLC25A1 can decrease the activity of the PPARγ signaling pathway, thereby reducing the expression of gluconeogenesis genes and improving hyperglycemia and glucose intolerance (37, 38). The protein Citrin, encoded by the SLC25A13 gene and expressed in hepatocytes, is involved in the exchange of citrate and aspartate. Patients with Citrin deficiency or adult-onset type II citrullinemia are prone to fatty liver, non-alcoholic steatohepatitis (NASH), and even hepatocellular carcinoma (37).

Type 2 diabetes mellitus (T2DM) is a metabolic disorder caused by energy overload, characterized by insulin resistance, hyperglycemia, and hyperinsulinemia. SLC proteins play a crucial role in the progression of T2DM by regulating glucose uptake, reabsorption, and utilization in peripheral tissues, as well as glucose synthesis in the liver, kidneys, and intestines. The involvement of SLC proteins in T2DM has been extensively studied. The SLC5 family (including sodium-glucose cotransporter 1 and 2) is involved in glucose reabsorption in the kidneys and glucose absorption in the intestines. The sodium-glucose cotransporter 2 (SGLT2), encoded by the SLC5A2 gene, is a currently approved drug target for the treatment of T2D (39–41). The GLUT proteins of the SLC2 family primarily control the transmembrane transport of glucose, maintaining stable blood glucose levels. GLUT2 is expressed in the basolateral membranes of intestinal and renal absorptive epithelial cells and is a potential target for diabetes prevention and treatment (42, 43). The SLC7 family (e.g., ASC-1) promotes the uptake and accumulation of serine in adipocytes, thereby inhibiting oxidative stress and insulin resistance. ASC-1 regulates lipid synthesis by enhancing serine uptake and protects adipocytes from oxidative stress and insulin resistance. Studies have also shown that the expression of SLC7A1 is closely related to the antioxidant capacity of adipocytes. Its absence can lead to increased oxidative stress and exacerbated insulin resistance in adipocytes (44, 45). The SLC13A5/sodium-coupled citrate transporter is associated with the pathogenesis of T2D and non-alcoholic fatty liver disease (NAFLD). Recent preclinical studies have demonstrated its potential for the treatment of T2D and NAFLD (46, 47). SLC30A8 (ZnT8) is a key zinc transporter in pancreaticβ-cells, regulating zinc homeostasis and thereby affecting insulin secretion. Genetic variants of SLC30A8 are associated with an increased risk of type 2 diabetes mellitus (T2DM). Using a tetracycline - inducible HEK293 cell line and FluoZin - 3 technology, researchers first revealed the partial plasma membrane localization of SLC30A8 and developed a method to assess its functionality. This provides a new approach for developing innovative diabetes therapies targeting zinc homeostasis (48).

The involvement of SLC proteins in the development of obesity has also attracted attention, particularly those transporters related to fatty acid oxidation and energy metabolism. The carnitine/acylcarnitine translocase (CACT), encoded by the SLC25A20 gene, is involved in the β-oxidation phase of fatty acids. Patients with CACT deficiency are prone to severe metabolic disorders, mainly due to impaired fatty acid oxidative metabolism (49–51). The APC1 protein, encoded by the SLC25A24 gene, is widely distributed in the body and facilitates the exchange of adenosine nucleotides. Mice with SLC25A24 gene knockout exhibit resistance to obesity caused by a high-fat diet, characterized by reduced liver weight and decreased triglyceride deposition in the liver (50). Monocarboxylate transporter 1 (MCT1), a key member of the SLC family, mediates the transmembrane transport of lactate and pyruvate. Its functional impairment disrupts cellular energy supply and acid-base homeostasis by blocking lactate/pyruvate flux, while aberrant expression is implicated in multiple metabolic disorders (52–54).

### 3.3 The role of SLC proteins in tumor immunology

SLC proteins, through their roles in regulating metabolism, signaling pathways, and immune responses, influence tumor growth, progression, chemoresistance, and immune evasion in various cancers. These studies underscore the importance of SLC proteins as potential therapeutic targets. Future research should further explore their specific mechanisms and clinical significance, offering new strategies and directions for cancer treatment (**Table 2**).

**Table 2:** The role of SLC proteins in tumor immunology.

Cancer type	Key SLC proteins	Main functional mechanisms	Clinical significance
Gastric Cancer	SLC1A3 (EAAT1)	Activates PI3K/AKT pathway, promotes glucose metabolism and tumor progression	High expression correlates with poor prognosis; potential therapeutic target
Hepatocellular Carcinoma (HCC)	SLC50A1	Enhances glycolysis and DNA damage repair via METTL3-m6A regulation, driving drug resistance	High expression linked to chemoresistance; potential for combination therapy
	SLC1A5, SLC2A1/2, etc.	Regulates glutamine/glucose metabolism, ion balance (e.g., SLC39A6 zinc ions)	Multi-target metabolic inhibition strategy
Clear Cell Renal Cell Carcinoma (ccRCC)	SLC1A3, SLC1A5	Hypoxia-driven glutamine metabolism promotes proliferation/metastasis	Associated with invasiveness; targets hypoxia pathways
	SLC4A7	Modulates intracellular pH/ion balance, affecting chemoresistance	Novel target to overcome drug resistance
Pancreatic Ductal Adenocarcinoma (PDAC)	SLC1A4, SLC1A5	Increases alanine/glutamine uptake to fuel proliferation and chemoresistance	Metabolic intervention; enhances chemotherapy efficacy
	SLC7A5, SLC38A1	Activates mTORC1 pathway via leucine/glutamine uptake, promoting metastasis	mTOR pathway inhibition
Breast Cancer	SLC1A5	High glutamine uptake drives HER2+ and TNBC proliferation	SLC1A5 inhibition suppresses TNBC growth
	SLC7A11, SLC31A1	Regulates ferroptosis/autophagy (SLC7A11) and cisplatin resistance (SLC31A1)	Overcomes chemotherapy resistance
Ovarian Cancer	SLC7A11, SLC31A1	Modulates redox balance and platinum resistance	SLC7A11 high expression paradoxically linked to better prognosis but platinum resistance
	SLC22A18	Tumor suppressor; downregulation reduces survival	Restoration may inhibit tumor growth
Glioblastoma (GBM)	SLC1A5	Polarizes TAMs to M2 phenotype, facilitating immune evasion	Targets tumor microenvironment
	SLC43A3	Oncogenic role in proliferation/migration	Potential combination with immunotherapy
Colorectal Cancer	SLC5A8, SLC22A18, etc.	Tumor-suppressive effects (e.g., SLC26A3 inhibits growth)	Restoration or targeted therapy
	SLC7A5, SLC39A4	Activates resistance-related pathways	Overcomes chemoresistance
Lung Cancer	SLC7A5	Leucine uptake activates mTORC1, promoting chemoresistance	mTOR pathway targeting
	SLC39 family, etc.	Regulates amino acid/metal ion metabolism	Multi-target combination therapy
Melanoma	SLC27A1 (FATP1)	Enhances fatty acid uptake/metabolism, driving invasiveness	Novel strategy targeting lipid metabolism

SLC proteins promote rapid proliferation of tumor cells by regulating the uptake of key nutrients and activating pro-growth signaling pathways. The SLC1 family of glutamate/glutamine transporters plays a significant role in various cancers. SLC1A3 (EAAT1) activates the PI3K/AKT pathway in gastric cancer, promoting tumor growth, with high expression correlating with poor prognosis (55). In ccRCC, it regulates glutamate levels under hypoxia, driving cell proliferation (56). SLC1A4 upregulation in PDAC enhances alanine uptake, supporting rapid tumor cell proliferation (57). SLC1A5 (AfSCT2) activates mTORC1 via glutamine uptake in HCC, driving tumor progression (58), and promotes glutamine uptake in ccRCC, linked to invasion/metastasis (59, 60), while in breast cancer (TNBC), it supports proliferation via glutamine metabolism (61). The SLC2 family of glucose transporters, including SLC2A1/SLC2A2, enhances glycolysis in HCC, promoting tumor growth (58), and increases glucose uptake in PDAC to fuel proliferation (62). In lung cancer, they regulate metabolic demands, influencing tumor progression (63). The SLC43 family, including SLC43A3 in GBM, has an oncogenic role, with high expression promoting proliferation/migration (64).

SLC proteins are involved in the metabolic reprogramming of tumor cells by regulating the uptake of metabolites such as amino acids, fatty acids, and metal ions. The SLC7 family, including SLC7A5 (LAT1), mediates leucine uptake in PDAC, activating mTORC1 and mediating chemoresistance (65, 66). In breast cancer, high expression is linked to metabolic reprogramming and drug tolerance (67–69), while in lung cancer, mTORC1 activation drives chemoresistance (63, 70, 71). SLC7A11 (xCT) in breast cancer regulates cystine/glutamate exchange, influencing ferroptosis and drug sensitivity (67–69), and in ovarian cancer, it is associated with cisplatin resistance via redox balance (72). The SLC27 family, such as SLC27A1 (FATP1) in melanoma, stimulates fatty acid uptake/metabolism, enhancing invasiveness (73). The SLC38 family, including SLC38A1 in PDAC, enhances glutamine uptake, promoting metastasis/invasion (74, 75). The SLC50 family, such as SLC50A1 in HCC, promotes chemoresistance via glycolysis/DNA repair, regulated by METTL3-mediated m6A modification (76).

Multiple SLC proteins are involved in chemoresistance mechanisms in tumor cells by regulating drug uptake, metal ion homeostasis, or metabolic pathways. In PDAC, SLC1A5 mediates chemoresistance via glutamine uptake (61). The SLC4 family, such as SLC4A7 in ccRCC, modulates intracellular pH/ion balance, impacting chemoresistance and invasiveness (56). SLC7A2 downregulation in ovarian cancer is linked to proliferation and chemoresistance (77). The SLC31/SLC39 families, such as SLC31A1 in breast cancer, correlate with cisplatin resistance (78), and in ovarian cancer, mediate cisplatin resistance (79). SLC39A4 in colorectal cancer promotes chemoresistance via metal ion regulation (80), and in lung cancer, activates signaling pathways driving chemoresistance (63). SLC39A6 in HCC promotes tumor progression through zinc regulation (81), and in lung cancer, influences metabolism and proliferation (63). SLC39A7 in lung cancer contributes to chemoresistance through metal ion homeostasis (63).

Certain SLC proteins contribute to tumor immune evasion by modulating immune cell metabolism or signaling pathways. In GBM, SLC1A5 modulates glutamine metabolism in TAMs, facilitating immune evasion (64). The SLC15/SLC46 families transport bacterial peptidoglycans to activate NOD1/2 in HCC, modulating tumor immunity (21, 26). The SLC30 family, including SLC30A8 (ZnT8) in HCC, regulates zinc homeostasis, which affects insulin secretion and indirectly influences the tumor microenvironment through genetic variants linked to T2DM risk (48).

Certain SLC proteins exhibit tumor-suppressive effects in specific cancers, and their downregulation is associated with tumor progression. The SLC22 family, such as SLC22A18, acts as a tumor suppressor in ovarian cancer, with downregulation correlating with reduced survival (78), and suppresses tumor growth in colorectal cancer when downregulated (80, 82, 83). The SLC26 family, including SLC26A3, has tumor-suppressive effects in colorectal cancer when downregulated (80, 82, 83). The SLC40 family, such as SLC40A1 in colorectal cancer, has tumor-suppressive effects when downregulated (80, 82, 83).

Some SLC proteins also influence tumor development through the regulation of ion homeostasis and signaling pathways. SLC26A4 in HCC regulates ion balance, promoting tumor progression (58). SLC26A6 in HCC modulates ion balance and metabolism (58), and in lung cancer, it influences metabolic signaling pathways (63).

### 3.4 The role of SLC proteins in neurological diseases

The SLC protein family plays a key role in the onset, progression, and management of neurological diseases. These findings provide a crucial scientific basis for a comprehensive understanding of the functions of SLC proteins in the nervous system.

SLC12A2 (NKCC1) promotes the depolarizing response of γ-aminobutyric acid (GABA) by mediating chloride ion uptake, and its altered activity balance is closely associated with epilepsy. Its inhibitor, bumetanide, has been proven to possess antiepileptic capabilities. Bumetanide inhibits NKCC1, reducing chloride ion influx and restoring GABAergic inhibition, thereby exerting an antiepileptic effect. SLC12A5 (KCC2) is the major chloride ion extrusion transporter, and its activity balance changes with SLC12A2 are highly related to epilepsy. The function of KCC2 is to expel chloride ions from the cell, maintaining a low intracellular chloride ion concentration and enhancing GABAergic inhibition (84). SLC1A2 (GLT-1) is a key component of the glutamate/GABA-glutamine cycle, and its mutations can cause impaired glutamate clearance, potentially leading to epilepsy. GLT-1 is primarily responsible for clearing glutamate from the synaptic cleft, maintaining low glutamate concentrations and preventing excitotoxicity caused by glutamate (85). Mutations or functional impairments of these SLC proteins are closely related to the occurrence of epilepsy, identifying potential therapeutic targets for epilepsy treatment. Future research needs to further elucidate the specific mechanisms of these SLC proteins and explore their application significance in epilepsy treatment.

In the brain tissue of Alzheimer’s disease (AD) patients, the expression of SLC2A1 is significantly reduced, inversely correlated with Aβ deposition. The decrease in GLUT1 likely affects glucose metabolism in the brain, thereby influencing the clearance of Aβ (86). SLC2A3 deficiency is associated with the hyperphosphorylation of tau protein, especially after the activation of astrocytes in AD patients (87). Elevated levels of SLC2A2 are observed in AD patients, which may be related to the overactivation of astrocytes and subsequent disruption of glucose metabolism in the brain (87). Reduced levels of SLC1A2 are linked to impaired glutamatergic homeostasis, potentially exacerbating glutamate excitotoxicity and affecting neuronal health (87). Variations in the SLC25A12 gene may lead to mitochondrial dysfunction, thereby affecting neuronal health (87). Increased expression of SLC16A4(MCT4) in the cerebrospinal fluid of AD patients is associated with cognitive decline. Overexpression of MCT4 may lead to increased neuronal apoptosis, thereby affecting cognitive function (88). These findings have identified potential therapeutic targets for AD treatment. Future research will further elucidate their specific mechanisms and explore their potential clinical applications.

SLC6A3 (also known as DAT, the dopamine transporter) is a crucial pathway for diagnosing Parkinson’s disease (PD). Using imaging techniques such as positron emission tomography (PET) and single-photon emission computed tomography (SPECT), the actual density of DAT can be detected, thereby assessing the integrity of dopaminergic neurons. Reduced DAT is closely associated with the progression of PD (89). Dysfunction of SLC6A3 leads to the accumulation of dopamine in the synaptic cleft, thereby disrupting normal neural signaling pathways and causing the symptoms of Parkinson’s disease (90). SLC7A5 facilitates the rapid entry of l-DOPA into the brain, assisting in dopamine synthesis and thereby improving PD-related symptoms (91, 92). These findings have highlighted potential targets for the diagnosis and treatment of PD. Future research will further explore their specific mechanisms and clinical application value.

SLC7A5 regulates the transport of branched-chain amino acids (BCAAs) and thereby disrupts the amino acid balance in the brain, affecting the efficiency of neural signaling. SLC7A5 plays a crucial role in the blood-brain barrier (BBB) by regulating the transport of BCAAs. Dysfunction of SLC7A5 can lead to abnormal levels of BCAAs in the brain, thereby disrupting neural signaling and potentially causing autism spectrum disorders. Studies have also found that intracerebral injection of BCAAs can improve abnormal behavioral phenotypes in mutant mice (93). Variations in the SLC19A1 gene are associated with childhood autism. Dysfunction of the vitamin B12 transporter encoded by SLC19A1 may lead to abnormal vitamin B12 metabolism and thereby interfere with normal neural development (94). Variations in the SLC19A1 gene are associated with childhood autism. Dysfunction of the vitamin B12 transporter encoded by SLC19A1 may lead to abnormal vitamin B12 metabolism and thereby interfere with normal neural development (95, 96). These findings provide potential targets for the diagnosis and treatment of autism. Future research will further elucidate the specific mechanisms and explore their clinical application value.

SLC proteins are also closely related to the pathogenesis of many other neurological diseases, such as attention deficit hyperactivity disorder (ADHD), intellectual disability, Huntington’s disease, and major depressive disorder. Drugs and inhibitors targeting SLC proteins, such as UCPH-101, tiagabine, and newly developed SLC18A2 inhibitors like deuterated benzoic acid and tetrabenzoic acid, have been used to treat these related diseases.

### 3.5 Research on SLC proteins in transplant immunology

Significant progress has been made in understanding the fundamental mechanisms of transplant immune responses, overcoming transplant rejection, and inducing transplant immune tolerance through research on SLC proteins. There is considerable inter-individual variation in the response to the commonly used immunosuppressant tacrolimus, which is influenced by genetic polymorphisms of SLC transporters. SLCO1B3 is localized to the basolateral membrane of hepatocytes and is primarily responsible for transporting tacrolimus into liver cells, thereby regulating its excretion (97).

SLC proteins also regulate the function and metabolism of immune cells, which are critical for transplant-related immune responses. GLUT1 and GLUT3 enhance glucose uptake to maintain T cell activation and promote their differentiation (97). CD4+ and CD8+ T cell subsets utilize SLC15A2 and MCT1 (SLC16A1), respectively, to take up large amounts of lactate. Glutamine cotransport mediated by SLC1A5 or SLC38A1, as well as leucine exchange mediated by the SLC7A5–SLC3A2 complex (CD98), promote mTORC1 activation through multiple mechanisms, thereby regulating T cell metabolism and the differentiation of Th1 and Th17 subsets (97). Different SLC proteins, through their transported substrates or as specific functional modules, influence various biological processes and events in dendritic cells, such as antigen presentation and the secretion of cytokines, chemokines, and granzymes. SLC proteins can rapidly sense damage signals and establish nanotube-based connections with resident phagocytes, thereby transferring mitochondria to nearby activated macrophages. This process is regulated by the pattern recognition receptor TRPM7 on SLCs. Mitochondrial proliferation is beneficial for suppressing the inflammatory properties of macrophages and regulating the immune response (97).

**Figure 1:**
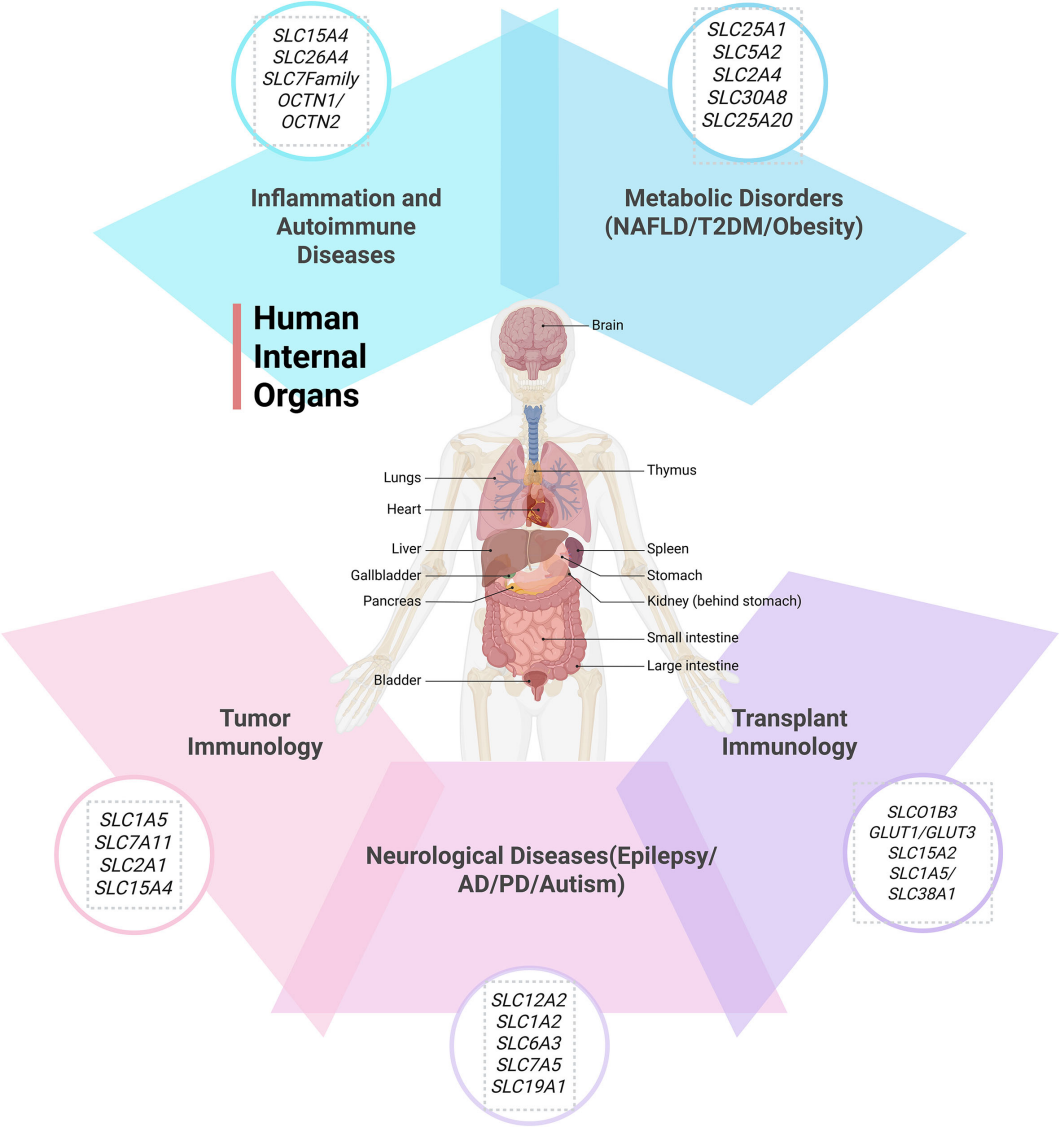
The roles of SLC family in various immune diseases (Created with BioRender.com).

These research findings indicate that SLC proteins play a key role in transplant immunology by regulating immune cell functions and signaling pathways. Future research has the potential to further elucidate the specific mechanisms of SLC proteins in transplant immunology, thereby providing a theoretical basis for the development of new immunotherapeutic strategies. These studies offer a crucial scientific foundation for a comprehensive understanding of the mechanisms of transplant immunology and the innovation of therapeutic strategies (**Figure 1**).

## 4 Pharmacotherapy and synergistic applications of SLC family

The SLC transporter family is responsible for the transmembrane transport of amino acids, nucleotides, sugars, fatty acids, inorganic ions, and drugs. These proteins are critical determinants of pharmacokinetics, pharmacodynamics, and toxicity of drugs. Alterations in the expression and function of SLC transporters can affect the pharmacokinetics, pharmacodynamics, and toxicity of the transported substances, thereby leading to drug-drug or drug-food interactions. The synergistic use of SLC proteins may offer new strategies for overcoming drug resistance and improving therapeutic outcomes.

### 4.1 Cancer

Under different culture conditions, inhibiting SLC2A5 (GLUT5) can affect the activity and proliferation of specific tumor cells. SLC2A5 promotes tumor cell metabolism and proliferation by increasing fructose uptake (98, 99). SLC13A3 is a sarcosine transporter in tumor cells that confers resistance to ferroptosis and undermines the efficacy of tumor immunotherapy. Tumor cells take up itaconic acid from tumor-associated macrophages (TAMs) via SLC13A3, activating the NRF2-SLC7A11 pathway to evade ferroptosis mediated by the immune system and develop resistance to immune checkpoint blockade therapy. Inhibiting SLC13A3 can enhance T cell-mediated immune responses, break through the macrophage-mediated immunosuppressive barrier, and inhibit tumor growth and metastasis (100, 101). In patients with pancreatic ductal adenocarcinoma, inhibiting SLC4A4 can lead to the accumulation of HCO_3_⁻ in the extracellular space and reduce lactate production by cancer cells, thereby alleviating acidosis in the tumor microenvironment (TME). The combination of SLC4A4 targeting and immune checkpoint blockade can overcome resistance to immunotherapy and extend patient survival (99). SLC1A1 promotes tumor development in lung cancer and NK T-cell lymphoma by regulating glutamine metabolism and R-2-HG levels. Targeting SLC1A1 can inhibit glutamine metabolism, reduce R-2-HG accumulation, and thereby suppress tumor cell proliferation while enhancing the efficacy of chemotherapy (102, 103).

SLC13A3 (a sodium-coupled citrate transporter) plays a key role in tumor immunity. Under the rubric of tumor immunity, SLC13A3 activates the NRF2-SLC7A11 pathway by taking up itaconic acid, thereby conferring resistance to ferroptosis in tumor cells and reducing the efficacy of immune checkpoint blockade (ICB) therapy. Inhibiting the activity of SLC13A3, or pharmacologically interfering with its function, can increase the sensitivity of tumor cells to ferroptosis, curb tumor progression, and enhance the effectiveness of ICB (104).

### 4.2 Metabolic diseases

SLC13A5 (also known as NaCT, a sodium-coupled citrate transporter) is highly expressed in the liver and is responsible for transporting citrate from the extracellular space into the cell. Citrate is a key energy sensor in cellular metabolism and is involved in processes such as glycolysis, the tricarboxylic acid (TCA) cycle, gluconeogenesis, and fatty acid synthesis. SLC13A5 plays a crucial role in metabolic disorders, such as obesity, insulin resistance, and non-alcoholic fatty liver disease (NAFLD). Its overexpression is closely associated with these metabolic disturbances. Inhibitors of SLC13A5 can reduce fat accumulation, enhance insulin sensitivity, and inhibit tumor cell proliferation. These findings provide a theoretical basis for the development of drugs targeting SLC13A5, which may potentially be used to treat these metabolic diseases. Future research will further elucidate the specific mechanisms of SLC13A5 and explore its clinical application value (105, 106).

SLC22A8 (OAT3) plays a crucial role in renal excretion, especially for the elimination of a large number of xenobiotics. With its broad substrate specificity, OAT3 regulates the renal excretion of various drugs and toxins. Steviol glucuronide (SVAG) is a substrate of OAT3, and inhibiting its transport activity may alter its pharmacokinetic properties (107–109).

## 5 Conclusions

This review summarizes the functions of the solute carrier protein family (SLC) in various diseases and their potential as therapeutic targets. The SLC protein family plays a crucial role in the transport of substances across cell membranes, regulation of metabolism, immune responses, and pharmacokinetics of drugs. The research findings highlight that SLC proteins have a significant impact on inflammation and autoimmune diseases, metabolic disorders, tumor immunology, and neurological diseases. To facilitate translational decision-making, we stratify SLC transporters into three evidence-based tiers: Tier A “validated” (n = 3)—SLC5A2 (empagliflozin), SLC2A1 (topical WZB117) and SLC47A1 (metformin substrate)—carry GWAS P < 5 × 10⁻^9^, functional validation in primary immune cells and marketed/phase III drugs, enabling immediate repurposing or indication-expansion trials; Tier B “candidates” (n = 3)—SLC7A5 (JPH203, phase II), SLC15A4 (CPI-455, phase I) and SLC1A5 (V-9302, phase I)—combine strong genetic and functional data with early clinical read-outs and now need safety profiling and predictive biomarkers; Tier C “potential” (n = 4)—SLC17A9, SLC16A1, SLC25A1 and SLC30A8—remain speculative, supported only by genetic or pre-clinical evidence and require chemical probe refinement, toxicology and first-in-human studies over the next 3–5 years. Thus, while the SLC super-family offers new targets for immune-related diseases, only tier A/B members presently represent clinically translatable strategies; tier C and ungraded members must await deeper mechanistic insights, and continued expansion of genotype–phenotype databases plus AlphaFold2-enabled structural biology is expected to upgrade further transporters, ultimately delivering precision immuno-metabolic therapy and genotype-guided individualized transplant immunosuppression. As our understanding of SLC protein functions and genetic polymorphisms deepens, there is hope for the implementation of more precise drug treatment regimens, particularly in immunosuppressive therapy following organ transplantation.
